# Dietary Factors in the Etiology of Parkinson's Disease

**DOI:** 10.1155/2015/672838

**Published:** 2015-01-20

**Authors:** Zeynep S. Agim, Jason R. Cannon

**Affiliations:** School of Health Sciences, Purdue University, 550 Stadium Mall Dr., West Lafayette, IN 47907, USA

## Abstract

Parkinson's disease (PD) is the second most common neurodegenerative disorder. The majority of cases do not arise from purely genetic factors, implicating an important role of environmental factors in disease pathogenesis. Well-established environmental toxins important in PD include pesticides, herbicides, and heavy metals. However, many toxicants linked to PD and used in animal models are rarely encountered. In this context, other factors such as dietary components may represent daily exposures and have gained attention as disease modifiers. Several *in vitro, in vivo*, and human epidemiological studies have found a variety of dietary factors that modify PD risk. Here, we critically review findings on association between dietary factors, including vitamins, flavonoids, calorie intake, caffeine, alcohol, and metals consumed via food and fatty acids and PD. We have also discussed key data on heterocyclic amines that are produced in high-temperature cooked meat, which is a new emerging field in the assessment of dietary factors in neurological diseases. While more research is clearly needed, significant evidence exists that specific dietary factors can modify PD risk.

## 1. Introduction

Parkinson's disease (PD) is the second most common neurodegenerative disease with a prevalence of ~1% among those over 60 years of age. PD is characterized by dopaminergic neuron loss in the substantia nigra followed by striatal dopamine depletion, which results in cardinal motor symptoms such as bradykinesia, postural instability, resting tremor, and rigidity. ~10% of PD cases are caused by genetic factors: mutations in the alpha-synuclein, Parkin,* PINK*,* LRRK2*, and other genes [1]. However the remaining ~90% of patients are sporadic, arising from unknown causes. Environmental factors are thought to play a crucial role in progression of the disease. Pesticide exposure has been repeatedly linked to PD [2]. Rotenone and paraquat have been shown to induce dopaminergic neuron loss in the substantia nigra and striatum in animals, resulting in development of PD-like symptoms [3]. Further, these pesticides have been linked as PD risk factors in humans. Another very well-known PD-causing toxin is 1-methyl-4-phenyl-1,2,3,6-tetrahydropyridine (MPTP). MPTP's dopaminergic toxicity was discovered after hospitalization of several people who were synthesizing homemade opioid drugs in early 1980's. MPTP is metabolized into MPP+ after crossing blood-brain-barrier. MPP+ is transported into dopaminergic neurons via dopamine active transporter and interferes with complex I activity in mitochondria, eventually increasing reactive oxygen species production and oxidative stress in the cell [3].

Although rotenone, paraquat, MPTP, and other toxicants have been repeatedly used to model PD, exposure to these specific compounds does not likely account for a significant number of PD cases due to relatively rare exposures in most environments. The search for compounds that are encountered frequently and exposed throughout the life is still ongoing. Traditionally, examination of dietary factors in PD has received less attention compared to other environmental exposures. However, several dietary habits have been shown to modify the risk of developing PD. Here, we critically review findings on the association of dietary groups, vitamins/antioxidants, metals, and fat, with modifying PD risk. The role of industrial and agricultural contaminants in PD has been reviewed numerous times [2, 4, 5], the focus of this review is on natural components of the diet, or compounds formed during preparation.

## 2. Vitamins

### 2.1. Vitamin A and Carotenoid

Carotenoids (alpha- and beta-carotene) are precursors of vitamin A in human. Egg yolks, organ meats, and milk are rich sources of vitamin A, while carotenoid rich diet includes carrots, sweet potatoes, and peaches, as well as other fruits and vegetables. Previously, vitamin A and beta-carotene were shown to inhibit alpha-synuclein fibril formation and destabilize formed fibrils in dose-dependent manner* in vitro* [6]. While several human studies did not identify a link with vitamin A and PD [7–10], Miyake et al. found a protective effect of beta-carotene in PD in a Japanese population [11]. In this study, dietary habits of 249 PD patients were compared with 368 controls. Beta-carotene at highest quartile (>4080.9 *μ*g/day) was inversely associated with PD [odd's ratio (OR) 0.56, 95% confidence interval (CI) 0.33–0.97]. Although there is no recommended daily allowance for beta-carotene due to lack of evidence, one study reported that daily intake range is 2–7 mg for women in US [12]. When data was stratified by sex, beta-carotene consumption remained significant only in women (*P* value = 0.001). Thus, current data on a role for vitamin A in PD development is extremely limited, with quantifiable effects only at the highest doses.

### 2.2. Vitamin B

Vitamin B complexes are found in meat, fish, cereal, dairy products, and some vegetables (i.e., potato) and fruits (i.e., banana). Although there are several types of vitamin B, the focus of this discussion is on vitamin B2 (riboflavin), B6 (pyridoxine), B9 (folate), and B12 (cobalamin).

Homocysteine is a metabolite of methionine that is essential for the DNA synthesis and has been shown to exert adverse effects of mitochondrial alterations. Vitamins B6, B9, and B12 indirectly regulate level of homocysteine [13, 14]. Folate-deficient diets result in increases in homocysteine [15]. High homocysteine level damages DNA and depletes energy reserves, subsequently inducing neuron apoptosis [16, 17].

In one study on the effects of folate deficiency, two-month-old C57B1/6 mice were subjected to a diet lacking folate or control diet containing 2 mg folate/kg of food for two months followed by intraperitoneal (ip) MPTP injection at subtoxic doses or saline [15]. Mice fed with control diet did not exhibit differences in motor activity between MPTP or saline groups. Similarly, motor activity in folate-deficient mice was not significantly different from mice with control diet. However, MPTP-induced motor activity impairment and loss of nigral dopaminergic neurons were exacerbated in folate-deficient mice. Further, vitamin B2 deficiency in rodents was shown to decrease circulating iron levels and increase iron turnover, resulting in disturbance of iron metabolism, which is one of the well-established hypotheses in PD [18, 19]. Therefore, in animal models, vitamin B deficiency appears to exacerbate neurotoxicant-induced motor deficits and pathology.

Epidemiological studies presented variable findings. Higher intake of vitamins B6, B9, and B12, but not B2, was associated with lower risk of PD in a German population [20]. A Rotterdam study that examined 7,983 individuals found that while vitamin B6 was protective against PD in dose-dependent manner (only in smokers), vitamins B9 and B12 were not significant [21]. It is not clear whether higher vitamin B6 intake prevents or simply delays PD. Given that significance was only achieved when combined with another known PD protective factor (smoking), there is currently no evidence that B6 alone would modify PD etiology. Lower intake of folate was detected in 249 PD patients, compared to 368 healthy controls, but the association was not significant after adjustment for potential dietary confounding factors [22]. The loss of significance after adjusting for multiple factors illustrates the difficulty in identifying single disease modifying dietary factors in human studies. In another study, Coimbra and Junqueira reported low levels of riboflavin in 31 PD patients [23]. Riboflavin supplementation (30 mg) with 8-hour intervals for 6 months gave rise to promising improvements in motor activity of patients. The protective effect remained intact, even when supplementation was stopped. In contrast, data from the Honolulu Heart Study (HHS), with 30 years of followup of approximately 8,000 men from Japanese-Okinawan ancestry reported that total vitamin B intake was not significantly associated with PD (using 137 patients) [9]. This study has utilized extensive data collection of patients' dietary habits and any environmental toxicant exposures over decades, such as pesticides, resulting in significantly more power compared to retrospective case-control studies. In two large cohort studies entitled “Nurse Health Study (NHS)” and “Health Professionals Follow-up Study (HPFS)” that contain 121,700 females and 51,529 males, respectively, average folate intake was determined as 482 *μ*g/day in men and 366 *μ*g/day in women, where folate levels were not significantly different between PD patients and healthy controls [24]. The questionnaire was used to assess daily consumption of particular food for last 12 months. No association was detected between vitamins B6, B9, and B12 and PD in US population [25]. One possibility is that folate might be protective only in neurotoxin-induced PD models, when administered at high doses, often prior to the neurotoxic insult.

### 2.3. Vitamin C

Numerous studies have suggested that there is no clear association between vitamin C and human PD [9–11]. Vitamin C was found to increase dopamine synthesis in human neuroblastoma cell line SK-N-SH [26]. Although vitamin C is the most potent antioxidant among other vitamins, exogenous administration may not affect disease development due to limited access to the brain. Access to the brain is restricted by high water solubility and the requirement of active transport at the choroid plexus to enter the brain [27].

### 2.4. Vitamin D

Major vitamin D rich foods are fortified milk, liver, and saltwater fish [28]. Vitamin D is metabolized into its active form, 1,25-dihydroxyvitamin D (1,25-(OH)2 Vit D or calcitriol) in the cytoplasm of neurons and glial cells [29, 30]. The vitamin D receptor (VDR) is activated by binding to calcitriol which increases calcium uptake in bones.

Although a protective role of vitamin D against PD is not well established, there are number of laboratory studies suggesting that exogenous administration may be protective: MPP+ toxicity in primary mesencephalic dopaminergic neurons was decreased by low doses of vitamin D (1–100 nM)* in vitro* [31]. Pretreatment of rats with calcitriol prior to 6-hydroxydopamine (6-OHDA) administration attenuated neuronal toxicity* in vivo* [32]. It is worth noting that significantly lower bone mass index and vitamin D deficiency were detected in PD patients [33]. Further, risk factors for hip fracture and falling in PD patients were associated with lower vitamin D plasma levels [34]. Treatment of a 47-year-old male PD patient with very high dose of vitamin D (4000 IU daily) with ongoing conventional therapy delayed tremor and rigidity, while other Parkinsonism symptoms did not show any alterations [35]. This report represents a single case and more additional robust studies have either not been conducted or have failed to show a direct protective effect of vitamin D in PD. A recent systemic review and meta-analysis included seven studies with 1,008 PD cases and 4,536 controls [36]. Statistical analysis indicated a ~twofold increase in risk of PD in individuals with vitamin D-deficient diet [OR 2.2, 95% CI 1.5–3.4]. Further research on association of vitamin D with PD needs to be performed. In particular, more robust epidemiological studies need to be conducted. Distance from the equator has been linked to prevalence of other disorders such as multiple sclerosis. While there have been many hypotheses to account for this relationship, sunlight exposure and vitamin D levels are plausible factors [2]. Given the studies mentioned above, PD prevalence and progressions rates along with distance from the equator and vitamin D levels should be assessed [37].

Examinations of genetic polymorphisms in the VDR as a factor in modulating PD risk or disease development have produced variable results [38]. The rs4334089 polymorphism in the receptor gene has been found significantly associated with PD in a US population study including a discovery phase of 770 Caucasian families with PD history and a validation case-control study (267 cases, 267 controls) [39]. However, the same polymorphism was not found associated with PD in Chinese Han [40] and Taiwanese populations [41].

### 2.5. Vitamin E

Vitamin E is found at high levels in vegetable oils, nuts, and whole-grain products. It has strong antioxidant capacity. Pretreatment of neurons with vitamin E alleviated MPTP-induced dopaminergic neuron toxicity* in vitro* [42]. Further, vitamin E-deficient mice exhibit heightened sensitivity to MPTP [43–45].

Association of vitamin E with PD has been identified in a number of studies in the last two decades. Serum levels of vitamin E in PD patients were significantly lower than controls [46]. One of the first studies compared frequency of early-life consumption of 31 foods in 106 PD patients and their spouses [47]. This study found that PD patients are less likely to eat vitamin E-containing foods (peanut and salad dressing) than those without PD. A few years later, a relatively large-sampled study was conducted using HHS data [48]. Here, all subjects were followed for 30 years and 24-hour recall for vitamin E-containing food was evaluated. Overall, this study showed an insignificant trend towards an association of high total vitamin E intake with decreased risk of PD. However, consumption of legumes was strongly protective. Unfortunately, the strength of these types of studies is limited by recall accuracy because 24-hour recalls potentially fail to reflect true dietary habits due to underreporting by some groups [9]. The primary reason why legumes are protective, but other foods containing high levels of vitamin E are not remains unclear. One possible explanation might be due to the presence of another unidentified compound that is present in legumes, but is absent or at low levels in other vitamin E rich foods.

Data from NHS and HPFS were used to assess dietary components over one year [8]. Female and male smoker PD patients were found to eat fewer nuts, which are rich in vitamin E content, while no difference was reported for mayonnaise and creamy salad dressings, contradicting the results of Golbe et al. [47]. However, it is difficult to conclude whether having the disease changed the food preferences or if nuts are really protective against PD. Differences in the lipid composition between vitamin E rich foods could also be contributing factors. Miyake et al. found that higher vitamin E intake (>9.759 mg/day) is significantly associated with decreased risk of PD in women (OR 0.33, 95% CI 0.15–0.71; *P* = 0.006) [11]. Interestingly, meta-analysis of eight epidemiological studies reported that moderate intake of vitamin E is protective with a relative risk of 0.81 [10]. Moreover, vitamin E supplementation has been tested as a therapeutic against PD in the DATATOP study [49]. In this study, 800 patients were supplemented with *α*-tocopherol, the biologically active component of vitamin E, for more than a year. No beneficial effect of *α*-tocopherol was observed during follow-up evaluation of PD symptoms. Vitamin E supplements contain racemic-*α*-tocopherol that has lower activity than RRR-*α*-tocopherol in foods [8, 50]. Thus, higher intake of supplementation might be needed to detect a protective effect. Also, penetration rates through blood-brain-barrier of these two vitamin E forms might be different. In addition, the use of vitamin E supplementation as a therapeutic is difficult to achieve due to the fact that more than half of dopaminergic neurons in substantia nigra of PD patients are already lost in the stage where symptoms are apparent [51].

In conclusion, the data for a protective or preventative role of vitamin E appears to be stronger than other vitamins. Further, low dietary levels could potentially increase risk. However, data on supplementation in patients already diagnosed with PD has failed to show a disease modifying effect.

## 3. Flavonoids

Flavonoids are the most common groups of polyphenols in human diet [52]. Many plant-based foods and beverages are rich in flavonoids, such as berry fruits and citrus fruits [53]. Flavonoids have high antioxidant capacity [54]; they have been shown to modulate oxidative-related enzymes and regulate mitochondrial function in neurons [52, 55]. These findings point to a potential protective role of flavonoids in PD.

Nobiletin, a flavonoid that is found in citrus fruit peel, was found to improve MPTP-induced motor and cognitive deficits in mice [56]. Although nobiletin administration (50 mg/kg) via ip injections for 2 weeks did not prevent loss of dopaminergic neurons in the midbrain of MPTP-induced PD model mice, motor deficits were alleviated significantly compared to mice that did not receive the injections.

A potential neuroprotective role of flavonoids in PD was recently examined using anthocyanins and proanthocyanidins. Strathearn et al. reported that the treatment of primary midbrain cultures with blueberry, grape seed, hibiscus, blackburrant, or Chinese mulberry extracts rescued rotenone-induced loss of dopaminergic neurons [57]. Here, blueberry and grape seed extracts were shown to rescue disruption of mitochondrial respiration, suggesting that the protective effect might be mediated via enhancement of mitochondrial function.

Epidemiological findings have also suggested that consumption of flavonoids in the human diet lowers PD risk. One study used NHS and HPFS datasets, which consist of 22.7 and 20 years of follow-up data, respectively, from 805 PD patients (438 men and 367 women) [58]. The major sources of flavonoids in this study were apples, tea, blueberry, strawberry, red wine, and orange/orange juice. Although the flavonoid intake was not significant in pooled PD incidents, men showed significant inverse association of flavonoid intake at highest quartile with PD [Hazard ratio (HR) 0.60, 95% CI 0.43–0.83]. The use of estrogen as a possible mechanism underlying gender difference was tested, but no association was found. Consumption of anthocyanin-rich fruits, strawberries, and blueberries reduced PD risk in pooled sample (HR 0.77, 95% CI 0.62–0.97). Another study, including 41 years of follow-up data from 2388 men and 2136 women in Finland, reported the association of berry consumption with increased risk of PD in men [relative ratio (RR) 1.80, 95% CI 0.85–3.82] [59].

One of the unavoidable controversies in epidemiological studies involving berries, as well as other fruits and vegetables, is the presence of pesticide exposure. Although, now many countries have strict regulations on use of pesticides and herbicides in agricultural areas, any exposure of fruits to pesticides could potentially reduce neuroprotective capacity. Another careful consideration will be to test subclasses of flavonoids separately. The specific content of flavonoids, such as flavanones and anthocyanins, varies between fruits. These subclasses exhibit differences in chemical properties and their ability to cross the blood-brain-barrier [60]. It is therefore possible that evaluation of fruit as one group might lead to misleading or weak associations.

## 4. Calorie Intake

Dietary restriction has been repeatedly shown to prolong lifespan and decrease age-related diseases [61–63]. Low calorie intake attenuated age-related decline in dopamine signaling and increased resistance of nigral neurons to excitotoxic or oxidative stress [64, 65]. Dietary restriction in mice enhanced expression of neurotrophic factor, especially brain-derived neurotrophic factor (BDNF), in hippocampus, resulting in an excitoprotective effect, preventing excitotoxicity which is caused by neurotransmitters such as glutamate [66–68]. Protein chaperones, such as hsp70, that help cells to resist various stressors, were also induced* in vivo* [69, 70].

In* C. elegans* treated with 6-OHDA, dietary restriction prevented dopamine depletion and dopamine cell loss, suggesting protective effect in anterior deirid and posterior deirid neurons [71]. Duan and Mattson (1999) have shown that vulnerability of dopaminergic neurons to MPTP was significantly decreased by lower calorie intake in adult male mice [69]. In another study, male Sprague-Dawley rats were subjected to low calorie diet for two or eight weeks and then injected intrastriatally with 6-OHDA [72]. Here, no differences on striatal dopamine terminal density or apomorphine-induced rotational behavior were observed in normal and restricted diet groups. It is possible that two months of low calorie diet might not be long enough to be protective against 6-OHDA toxicity in rats. A longer exposure to calorie-restricted diet was tested in rhesus monkeys [73]. Here, 13 adult rhesus monkeys were subjected to normal or calorie-restricted diet for 6 months followed by 2.4 mg MPTP injection through carotid artery. Behavioral assessment six weeks after MPTP injection revealed improved motor activity in calorie-restricted monkeys. Imaging and postmortem brain examination confirmed low calorie intake prevented MPTP toxicity, with decreased presynaptic dopamine loss and greater presynaptic dopamine activity in left and right basal ganglia measured using PET scan.

Epidemiological studies have suggested contradicting results on the protective effect of low calorie intake in PD. Low calorie intake is associated with reduced risk of PD [25]. In contrast, although individuals with lowest body-mass index experienced the lowest incidence of PD, the difference failed to reach significance level in Honolulu Heart Study [9]. In US, average calorie intake is 2,700 kcal for women and >3,000 kcal for men. It has been concluded that low calorie intake (1,600–2,000 kcal) beginning at approximately 20 years of age would have protective effect against PD [74].

## 5. Caffeine

Decreased risk of PD resulting from caffeine intake has been repeatedly identified in US, European, and Asian populations [75, 76]. Common sources of caffeine are coffee, tea (especially black tea), soft and energy drinks, and chocolate. Caffeine is known to be a central nervous system stimulant and an adenosine receptor antagonist [77, 78]. While adenosine receptor antagonists were shown to improve motor activity in MPTP administered primates [79, 80] and caffeine intake reduced MPTP-induced dopamine depletion in mice [81], agonist of the receptor disrupted dopamine transmission, resulting in exacerbated motor deficits in rodents [82].

There are number of case-control, cohort studies, and meta-analyses regarding caffeine intake and PD risk in different populations. Data from HPFS reported an inverse association between coffee (RR 0.5, 95% CI 0.1–2.1) and tea consumption (RR 0.6; 95% CI 0.3–1.2) with PD in very large population of males [83]. Interestingly, the Nurse Health Study that includes more than 120,000 female nurses did not find a significant inverse association, although a U-shaped dose-response curve between coffee intake and PD was observed, suggesting that individuals with moderate consumption of coffee (1–3 cups per day) had the lower risk of PD [84]. Another larger cohort study, HHS, gave different conclusions due to consideration of period of consumption. Grandinetti et al. reported that coffee intake decreased risk of PD, but it was no longer significant after adjustment for smoking [85]. A few years later, another study used the same HHS data, including more PD patients (102) and longer follow-up, and observed significant inverse association of coffee with PD, independent from alcohol and smoking habits [86]. Liu et al. also reported similar findings in a very large cohort study of US population [87]. Total caffeine intake was protective in dose-dependent manner also in a Chinese population (including 157 PD cases) and remained as a significant association after being stratified by smoking [88]. A strong protective effect was also observed in black tea consumption, as reported in other epidemiological studies in other Chinese and US studies [89–91], although no significant association was observed in green tea consumption [88].

It is noteworthy to mention that the inverse association of caffeine intake in women is not as straightforward as in men, a potentially major factor in the variable findings on the relation between the amount of coffee consumption and PD risk. In addition to the U-shaped relation between coffee intake and PD, implicating that the moderate consumption of coffee is neuroprotective against the disease [84], another large case-control study with 392 cases reported that only high coffee intake was significantly associated with lower risk of PD (OR 0.58, 95% CI 0.38–0.89) [92]. Rugbjerg et al. also reported the inverse association of highest coffee consumption, but not tea, with lower PD incidence [93]. In contrast, the relation was not significant in Finnish female population [94]. The contradicting results may be due to estrogen levels in women. Ascherio et al. proposed the association between coffee intake and estrogen use in two different studies. Higher caffeine intake in women who did not use hormone therapy was associated with a lower PD risk and mortality, while among estrogen users higher intake increased PD risk and mortality significantly [84, 95]. It is surprising that Liu et al. reported the opposite. The higher intake of coffee intake was associated with lower risk of PD among hormone users, but the association could not reach significance threshold [87]. Reconciling differences is a difficult task, especially given that the role of hormone use on neuroprotective mechanisms of caffeine is not known. It has been emphasized that the type of hormone, duration of use, and recipient's age affect the health outcome [96]. The relation between caffeine intake, estrogen use, and PD is difficult to explain and requires more careful consideration of case-control study design with discrepancies among hormone usage when dealing with female populations. The discrepancies described above illustrate the importance of considering influence of other potentially neuroprotective factors in PD such as smoking and estrogen.

## 6. Fatty Acid Intake

Total dietary fat intake is supplied in three categories: Saturated fatty acids, unsaturated fatty acids, and cholesterol. Cholesterol typically provides only 1% of fat intake, while the remaining 99% comes from fatty acids [21]. Cholesterol is found in animal products, such as meat, eggs, milk, and butter. Fatty acids are divided into two groups: saturated and unsaturated fatty acids. Dairy products and meat are rich in saturated acids. Monounsaturated fatty acids (MUFAs) are found in sunflower oil, peanut oil, and olive oil, which are commonly used in Mediterranean diet. There are different types of polyunsaturated fatty acid (PUFA): vegetable oils that contain omega-6 and omega-3 is abundant in fish and marine products.

Approximately 60% of structural material of the brain is composed of lipid. Synthesis of brain lipid requires essential fatty acids, suggesting that balance in dietary fatty acid intake is crucial for brain function [97–99]. PUFAs are involved in neural function and cerebral structure [100, 101]. Two types of omega-3 fatty acid, eicosapentaenoic acid (EPA) and docosahexaenoic acid (DHA), are important for lipid bilayer composition [102, 103]. In omega-3 deficiency, growth factors in brain, especially BDNF, fail to be produced [104]. Here, we have summarized findings on the effect of fatty acid intake in brain function and PD.

MUFAs and PUFAs have been shown to have anti-inflammatory and neuroprotective properties, by reducing the oxidative stress and inhibiting neuronal apoptosis [105–110]. PUFAs help regulation of dopamine activity in basal ganglia, controlling movement [111, 112]. Omega-3 fatty acid is crucial for neurogenesis in olfactory bulb and myelination by oligodendrocytes, which has been shown to be significantly affected in PD [113]. Moreover, omega-3 deficiency causes alteration of the dopamine mesocorticolimbic pathway, which is anatomically relevant to PD [114, 115]. Zimmer et al. reported that dopamine levels in cerebral areas and D2 receptor mRNA expression in frontal cortex were lower in rat fed with omega-3 deficient rats [115].

Supplementation or higher intake of unsaturated fatty acids was shown to alleviate neurotoxin-induced PD-like syndrome. Samadi et al. have shown that DHA reduced and delayed levodopa-induced dyskinesia in monkey [116]. A protective role of omega-3 fatty acid (120 mg EPA and 180 mg DHA) supplementation for three months has also been reported in 6-OHDA-lesion model of PD [117]. In another study, mice were supplemented with DHA (424 mg/kg) before seven MPTP ip injections at 20 mg/kg free base, which produces moderate dopamine denervation [118]. High DHA supplementation also prevented MPTP-induced decrease in dopamine in striatum and loss of tyrosine hydroxylase (TH)-positive nigral neurons. In contrast, in biophysical studies, PUFAs cause enhanced oligomerization of alpha-synuclein and insoluble alpha-synuclein aggregate formation and increased Lewy-like inclusions* in vitro* [119–121]. Studies with rodent models also reported that diet with high concentration of PUFA upregulated alpha-synuclein expression [122, 123]. Moreover, DHA-specific diet (0.69% DHA) increased alpha-synuclein accumulation in brainstem, followed by increased astrocyte activity in A53T (a disease-causing mutation) alpha-synuclein mice [124]. Given the key role that alpha-synuclein plays in sporadic and genetic PD cases, elevated PUFA diet studies need to carefully evaluate behavioral, biochemical, and especially pathological endpoints.

As the neuroprotective effect of PUFA has been repeatedly shown, both* in vitro* and* in vitro*, high fat diet (HFD) has been shown to be associated with increased risk of PD. HFD increases nigral dopaminergic neurons susceptibility to environmental insults [125]. For example, HFD fed rats (60% and 20% of calories from fat and carbohydrates, resp., compared to 10% calories from fat in regular diet) for 5 weeks were unilaterally injected with 6-OHDA into the medial forebrain bundle [126]. HFD rats showed enhanced striatal dopamine depletion and increased dopamine turnover in substantia nigra. A later study from the same group reported an increase in iron deposition, impaired dopamine function in dorsal striatum, and affected iron transport proteins in substantia nigra in HFD rats, suggesting the role of interplay between high fat intake and iron metabolism in PD [127].

Epidemiological studies have also suggested a protective role of unsaturated fatty acids in PD, while saturated fatty acids were associated with increased risk of developing the disease. Three large cohort studies using NHS, HPFS, and HHS data reported lower PUFA intake in PD patients compared to healthy controls [9, 20, 128–130]. Fish oil supplementation (180 mg EPA and 120 mg DHA for 3 months) had an antidepressant effect in PD patients with major depression symptoms [131]. Comparison of dietary habits of 89 PD cases and 336 healthy controls showed that high level of PUFA intake was protective in those exposed to paraquat [132]. The results on other types of fat intake are controversial. Total and animal fat intake was associated with increased risk of PD [7, 25, 133]; however, some studies reported no association with the disease progression [11, 20, 21, 128–130]. A very recent study analyzed fat intake of 1,087 PD patients and almost 300,000 controls from National Institutes of Health-American Association of Retired Persons Diet and Health Study [134]. Subclasses of fat (PUFA, MUFA, cholesterol, etc.), as well as overall fat intake, did not show any significant association with PD. Currently, it is the largest prospective analysis of dietary intake, considering types of fat intake separately. However, using an older population and having dietary assessment only at the baseline, rather than a follow-up report, has considerable limitations. Overall, it appears that PUFA intake may modulate PD risk. Diet and toxicant interaction studies are beginning to identify important interactions. Understanding the mechanistic bases of such interactions could potentially lead to new therapeutic approaches.

## 7. Metals

Exposure to metals can be due to different sources: occupational, dietary, or as contaminant in water or air. Occupations such as welders, smelters, and miners are at high risk of exposure to metals such as manganese and iron [135, 136]. Overall, occupational exposures to metals is typically higher in terms of dose, but rarer, compared to dietary consumption at high levels from supplementation or other sources. The route of exposure also needs to be considered, which is often inhalation in occupational settings.

### 7.1. Iron

Iron accumulation in PD has been studied for decades in imaging,* in vitro,* and* in vivo* studies. Iron accumulates more in substantia nigra of PD cases than it does in other brain regions [137]. The iron hypothesis in PD suggests that Fenton's reaction induces production of hydroxyl radical and higher oxidation states of iron [138–140]. Hydroxyl radicals are toxic to neurons by inducing lipid peroxidation and subsequent cell death. Lewy bodies in PD brains are iron-positive and iron has been shown to induce alpha-synuclein accumulation [141, 142]. Reactive microglia, a common pathological finding in PD brains, contain high levels of iron [143, 144].

Mice that received high iron diet were shown to be more vulnerable to environmental insults. Lan and Jiang have shown that mice fed with high iron diet for a month and received MPTP injection had lower levels of glutathione, higher levels of oxidized glutathione, enhanced formation of hydroxyl radicals and oxidized lipids, accompanied by loss of striatal dopamine and DOPAC in brainstem, compared to mice with control diet [145]. Abnormal iron intake exacerbates MPTP-induced toxicity* in vivo* [146] and enhances alpha-synuclein fibrillation in human BE-M17 neuroblastoma cells overexpressing A53T alpha-synuclein [147]. In contrast, iron-deficient rodents have shown impaired dopamine transport [148], decreased expression of dopamine receptor 1 and 2 in dose- and time-dependent manner [149–151], suggesting that balance in iron is needed for proper dopaminergic activity and TH activity. Therefore, although chelation of iron as a therapeutic approach was suggested with additional support that chelation of iron* in vitro* with desferoxamine prevents MPTP-induced cell death [152],* in vivo* efficacy was likely limited because of two major reasons: many chelators cannot cross blood-brain-barrier and they can deplete all iron and TH synthesis will also be inhibited [153].

A case-control study, which includes 126 PD cases and 432 controls from the Detroit area reported that higher dietary iron intake was significantly associated with increased risk of PD (OR 1.88, 95% CI 1.05–3.38) [25]. An interesting relation between iron, animal fat, and PD has been reported in a case-control study of New Yorkers [154]. Although dietary iron itself was not significantly associated with PD, the highest quartile of animal fat intake accompanied by low transferrin saturation level was very strongly associated with PD (OR 9.0, 95% CI 2.7–29.9). Transferrin level is an indicator of iron stores, suggesting that, in case of low transferrin levels, there are more free iron atoms to induce oxidative stress [155]. It is noteworthy that intake of particular type of iron through diet matters. Dietary iron can be presented in three types: (1) Heme iron (found in red meat and absorbed well by the human body), (2) nonheme iron (found in vegetables, such as spinach and in grain/cereal and not well absorbed by body), and (3) supplementation. Logroscino et al. (2008) reported that dietary iron intake is moderately associated with increased PD risk (RR 1.30, 95% CI 0.94–1.80; *P* = 0.02) in NHS and HPFS data [156]. While nonheme iron intake was related with increased risk of PD (RR 1.27), heme iron intake had no effect in disease progression. Iron supplementation was found related to PD only in men, suggesting a potential gender-specific metabolism of iron. In contrast, in a Japanese population, after being adjusted for several confounders (vitamin E, vitamin B6, caffeine, alcohol, cholesterol, smoking, sex, and age), higher dietary intake was protective against PD with a *P* value lower than 0.0001 [11]. This result suggests the interplay of iron with other dietary factors and importance of confounder effects. Fukushima et al. compared blood iron levels of PD patients and controls, as well as dietary habits [157]. It has been reported that dietary iron intake was not associated, but instead iron exposure via contamination of drinking water and airborne metals was more prominent in China. Finally, it should be noted that while many retrospective studies have identified links between dietary iron and PD, large highly powered prospective studies have failed to identify a convincing link [156].

Extra caution is needed to evaluate the cause of iron accumulation in the brain of PD patients. Iron uptake in the gut is highly regulated. Thus, genetic predisposition might also play an important role in iron accumulation in the brain. In two different genetic studies, PD patients were shown more likely to have polymorphism in transferrin [158] and hemochromatosis gene [159]. Neurotoxic models of PD, such as rotenone, disrupt iron homeostasis [160], suggesting that dietary iron intake, genetic factors, and environmental exposures, might play a combinatorial role in accumulation of iron in PD brains.

### 7.2. Manganese

Occupational manganese exposure at chronic and high levels in welders, miners, and smelters was associated with increased incidence of Parkinsonian-like symptoms [161]. A population-based case control study in Detroit also reported increased risk of PD with >20 years of manganese occupational exposure [162]. Besides occupational settings, manganese is a natural product of most of the foods: legumes, nuts, grains, some fruits, and vegetables. Different types of cereals and mixed nuts have high manganese levels, ranging from 20 mg/kg to 40 mg/kg [163]. However, the relation between dietary intake of manganese and PD is not very clear. Miyake et al. conducted a case-control study using Japanese PD patients and healthy controls and evaluated their dietary habits over a month [11]. In this study, dietary manganese intake did not affect PD risk after adjustments for confounders. In China, manganese exposure from nutrients was not associated with PD [157]. In another study, 250 PD patients and 388 controls from western Washington State participated in a case-control study to determine relation between dietary manganese with PD risk [130]. Although manganese intake alone was not significant, diets with low manganese and high iron or high manganese and high iron were significantly associated with increased risk of PD incidence (OR 1.2 and 1.9). These results are in agreement with a previous rat study [164], suggesting a potential synergetic effect of two heavy metals in the disease pathogenesis. Many foods are rich in both manganese and iron, including spinach, peas, nuts and seeds, which is a fact that further epidemiological studies should consider carefully.

### 7.3. Magnesium and Calcium

Magnesium is a cofactor for crucial processes in cell, such as protein and nucleotide synthesis, cell cycle activities, and mitochondrial integrity. It also modulates calcium and potassium transport via pumps, carriers, and channels [165].

Magnesium supplementation has been found to be protective in neurotoxicant-induced PD models [166, 167]. It also inhibits spontaneous and Fe-induced aggregation of alpha-synuclein [142]. Oyanagi et al. investigated the effects of calcium and/or magnesium deficiency over two generations in Wistar rats [168]. Rats that have received low magnesium diet (14 or 40 mg/100 g of food), compared to rats with control diet (70 mg magnesium/100 g of food), showed lower dopaminergic neuron count, more active microglia, and decreased fiber size of myelin fibers in substantia nigra. Dopaminergic neurodegeneration in the substantia nigra was more evident in magnesium-deficient rats than calcium and magnesium deficient rats. The same study also suggested that magnesium deficiency is toxic to the dopaminergic system only if occurring in fetal and newborn periods of life, suggesting the importance of magnesium in early development of dopaminergic neurons. The synergistic effect of calcium and magnesium was confirmed in a mouse model as well. Mice fed with low calcium/magnesium-deficient diet displayed cataleptic behavior, which was inhibited by treatment with L-DOPA in a dose-dependent manner, and these mice had significantly lower TH activity in substantia nigra [169]. Since magnesium was found to decrease NMDA activity [170], hypofunction of dopamine might be due to supersensitivity of NMDA receptors enhanced by lack of magnesium.

In humans, magnesium showed a protective effect against PD in a Japanese population (OR 0.33, 95% CI 0.17–0.73 for highest quartile >312.9 mg/day; *P* = 0.002) [11]. A relatively small-sized case control study in Sweden reported that lower dietary magnesium intake was associated with lower brief smell identification test score (*P* = 0.012), which is an olfactory function test in which PD patients generally perform less efficiently than healthy controls [171].

## 8. Alcohol

The association between alcohol consumption and PD risk has been investigated in several epidemiological studies. Most studies are adjusted for sex and age while some considered smoking, which is often concomitant with alcohol.

A small twin study consisting of 31 monozygotic twin pairs reported that the unaffected twin drank more alcohol than the affected twin (RR 0.5) [172]. A Spanish study (128 PD patients and 256 controls) showed that drinking more than 50 g/day of alcohol (*P* < 0.001) was inversely associated with PD in males, while the association was absent in female [173]. A crude inverse association of alcohol (beer, wine, and liquor) with PD was observed in a Swedish population, although the significance disappeared after adjustment for smoking [174]. Similarly, in Leisure World cohort study (395 cases and 2,320 controls from Southern California), PD risk was lower for drinkers of 2+ alcoholic drinks/day (OR 0.77, 95% CI 0.58–1.03) [175]. In contrast, an Italian case-control study reported that individuals with light to moderate alcohol drinking exhibited reduced PD risk compared to ones with heavy drinking or nondrinkers [176]. In another cohort study, in Swedish population, PD was associated with increased risk of alcohol use disorder (HR 1.3, 95% CI 1.25–1.53) with the highest risk in lowest age group, <44 years old (HR 2.39, 95% CI 0.96–5.93) [177]. To date, few studies have convincingly identified alcohol use as a significant risk factor for PD. A major strength of this study is that it analyzes 13–17 years of follow-up data of 1,083 PD patients hospitalized with an alcohol use disorder and 658 PD patients with appendicitis among 602,930 individuals in total. Although the datasets are rather large, the alcohol data was not adjusted for potential confounding factors, such as smoking. Another possible explanation why the findings of this study were inconsistent with previous findings might be due to their study design. Here, the alcohol consumption of the control was not known. The fact that PD patients admitted to hospital with appendicitis were not questioned for alcohol consumption becomes a significant limitation.

A meta-analysis was performed recently to investigate association of alcohol consumption with PD risk [178]. Meta-analysis of 32 studies including 9,994 cases among 677,550 subjects found a significant association of beer with decreased risk of PD (RR 0.59, 95% CI 0.39–0.90), but not with wine and liquor. In conclusion, the consumption of alcohol was associated with lower risk of PD in most of case-control and cohort studies, while the association generally disappeared after adjustment with smoking. Careful adjustment for age, smoking habit, caffeine intake, amount of alcohol, and types of alcohol, including compounds associated with specific alcoholic beverages (ex. tannins in wine) that have been consumed, needs to be performed in future studies.

## 9. Byproducts of Preparation

The discussion above focuses on several natural components of the diet. However, few human foods are consumed in the “raw” state. The preparation process itself may result in the formation of neurotoxicants.

Heterocyclic amines (HCA) were isolated and characterized in fried and broiled meat more than 30 years ago [179]. At that time, only five compounds were identified: 3-amino-l,4-dimethyl-5H-pyrido[4,3-b]-indole (Trp-P-1), 2-amino-9H-pyrido[2,3-b]indole (AaC), 2-amino-3-methyl-9H-pyrido[2,3-b]indole (MeAaQ), 2-amino-3,8-dimethylimidazo[4,5-f]quinoxaline (MelQx), and 2-amino-3-methylimidazo[4,5-f]quinoline (IQ). Since then, more than 20 HCAs were identified in cooked meat [180]. The amount of HCAs formed during the cooking process depends on time, temperature, and type of meat [181]. Several HCAs were identified as mutagens* in vitro* and* in vivo* studies, including in nonhuman primates [182–184].

Association of some HCAs with neurological disorders and neurotoxic mechanisms has been investigated. Two of those are Trp-P-1 and 3-amino-1-methyl-5H-pyrido[4,3-b]indole (Trp-P-2). Unilateral infusion of Trp-P-1 and Trp-P-2 in the rat striatum caused increase in dopamine and decrease in its metabolites in striatum, suggesting inhibition of monoamine oxidase [185]. Impairment in dopamine catabolism by Trp-P-1 and Trp-P-2 has been reported in the following studies [186, 187]. Moreover, Trp-P-2 was found to inhibit L-amino acid decarboxylase in human brainstem [188]. In the same study, the most potent inhibitors of dopamine synthesis after Trp-P-2 were identified as 2-amino-1-methyl-6-phenylimidazo[4,5-b]pyridine (PhIP) and Trp-P-1.

PhIP is a heterocyclic amine that may account for up to ~75% of the genotoxic material from the crust of cooked meat [189, 190], implicating that it can be consumed repeatedly and in high doses throughout the life. PhIP is highly mutagenic and is associated with several cancer types including breast, prostate, and colon [184]. Although PhIP and its N-hydroxylated metabolite, N-OH-PhIP were shown to cross blood-brain-barrier [191], their effects on nervous system were unknown. High dose PhIP administration caused tremor and freezing in female mice (personal communication with Drs. K. Turteltaub and A. Director-Myska). Recent neurotoxicity studies on PhIP* in vitro* using primary midbrain cultures have been performed [192]. PhIP and N-OH-PhIP treatments at 1 *μ*M were selectively toxic to primary dopaminergic neurons isolated from embryonic day 17 rat embryos. Here, dopaminergic neurotoxicity of PhIP and its metabolite were mediated by oxidative stress, which can be prevented by pretreatments with the antioxidant, N-acetylcysteine and compounds with antioxidant capacity, such as blueberry extract.

Harmane (1-methyl-9H-pyrido[3,4-b]indole) is another HCA, that is produced endogenously and is exposed exogenously from meat and other foods [193]. Blood levels of harmane were associated with essential tremor, which is one of the most common neurological diseases, characterized by action tremor of the hands [194, 195]. Case-control study including 150 essential tremor and 135 controls reported that blood harmane concentration was ~50% higher in cases than controls (OR 1.56, 95% CI 1.01–2.42) [196]. The same group was able to replicate the association in a separate case-control group from New York [197]. Recently, blood harmane concentrations were compared in 113 PD cases and 101 controls [198]. Blood harmane was significantly elevated in PD patients compared to healthy controls (OR 2.31, 95% CI 1.46–3.67).

The data from a limited number of studies on HCAs and PD suggests that these mutagenic compounds should be evaluated as dopaminergic neurotoxicants and etiological factors in PD. There are several gaps in the literature that need to be addressed. Future studies with HCA administration in animals will help to understand whether HCA exposure in animals can reproduce the clinical PD phenotypes. Moreover, epidemiological studies that investigate meat cooking time and doneness preferences of PD cases and controls will be very useful to reveal the association between HCA and PD.

## 10. Conclusion and Future Research

Parkinson's disease is a common neurodegenerative disorder, affecting individuals especially over 60 years of age. There is no known prevention or certain cure for the disease. Although rare mutations have been identified that cause familial PD, the majority of incidence remains as a mystery. For a long time, environmental factors have been a major concern related to disease pathogenesis. A multitude of environmental and occupational exposures have been implicated as PD risk factors. However, both increasing disease prevalence and the legislation reducing the use of many pesticides have renewed the search for the frequently encountered environmental factors that modify disease risk.

Here, in this review, we summarized key findings regarding the modulating effects of dietary factors in PD. The presence of contradicting findings on a single nutrient in the literature is a common problem. Especially, as discussed throughout the review, epidemiological studies might not have confirmed the findings from* in vitro* and* in vivo* studies. To solve this controversy, there are several points that need to be considered related to study design.

1. Epidemiological studies in the literature usually reported opposite findings on a single nutrient. Careful consideration is needed in the design of the study. A homogenous cohort or case-control population and large number of data will increase the chance of validation of results in further studies. In addition, while multivariate analysis including sex and age is essential, the adjustment for other dietary factors and habits (such as smoking) is highly recommended to increase the power of the study. Three such studies are HHS, NHS and HPFS, including approximately 8,000, 120,000 and 50,000 individuals, respectively. Patients' dietary habits, amount of food intake, and life styles have been followed and recorded for 20–30 years, creating very valuable datasets. Establishing more studies with large sample size and long follow-ups will prevent many crucial limitations in most epidemiological studies. Further improvement will be achieved by recruiting samples in early ages in life, instead of 30–50 years of age as in these three studies. By the time PD symptoms appear, significant nigral dopamine neuron loss has occurred. Testing dietary habits and intake of specific food groups in different stages of life, including early development and puberty, will also increase the strength of studies.* In vivo* studies utilizing chronic or even multigenerational studies on consumption levels of dietary factors will likely provide significant mechanistic insight.
2. The common limitation of epidemiological studies on dietary habits comes from the self-administered food questionnaires. PD patients may recall specific food consumption at a higher rate than controls in an effort to implicate risk factors. This is also known as* recall bias*. There are additional findings that having the disease might change food preferences [199, 200], suggesting that food surveys can lead the false results.
3. Investigation of dietary factors has inherent difficulties. A single food does not contain a single micronutrient. For example, dairy products are rich in vitamins A, B, and D, and also fat. A very recent meta-analysis included seven studies with more than a thousand PD patients among more than 300,000 individuals and identified dairy product as a risk factor for PD [201]. However, determination of which natural component or contaminant in these contributes to the association of the disease is important for early prevention and to identify mechanisms of pathogenesis.
4. The effect of combinations of food molecules needs to be considered. This type of approach is usually seen in combination of molecules from the same group: total vitamin intake and overall fat intake. However, evaluating composition of different groups of macronutrients is also crucial. For example, PUFAs are known to have a protective effect against neuroinflammation and oxidative stress, which are two common mechanisms in neurodegenerative diseases. Assessment of PUFAs along with vitamins and flavonoids, two large groups of antioxidants, might increase significance of their association with the disease. The traditional Mediterranean diet is defined with the high intake of olive oil, legumes, vegetables, and fruit, along with lower consumption of meat, poultry, and animal fats [202]. In a small case-control study, higher Mediterranean diet score was significantly associated with lower risk of PD [203]. Prospective and cohort studies with larger sample sets evaluating a particular diet or nutritional pattern are needed to understand dietary risk factors for PD.
5. Although genetic and environmental factors (pesticides, occupational exposures, and dietary habits) in PD have been investigated extensively, limited data on interactions are available. Genetic predisposition might greatly modulate the association of environmental factors with PD. A new advanced approach to genome-wide association studies investigates the effect of environmental factors in relation with polymorphisms or mutations in genomic level. A recent study followed this approach and showed combinatorial association of a polymorphism in glutamate receptor gene,* GRIN2A*, and caffeine intake in PD risk [204]. This study also independently validated findings of previous genome-interaction studies [205]. Future studies that consider more than one genetic and environmental factor in the risk of PD will generate more consistent findings and pave the way to reveal underlying mechanisms in the disease pathogenesis.

## References

[1] Singleton A. B., Farrer M. J., Bonifati V. (2013). The genetics of Parkinson's disease: progress and therapeutic implications. *Movement Disorders*.

[2] Cannon J. R., Greenamyre J. T. (2011). The role of environmental exposures in neurodegeneration and neurodegenerative diseases. *Toxicological Sciences*.

[3] Cannon J. R., Greenamyre J. T. (2010). Neurotoxic *in vivo* models of Parkinson's disease: recent advances. *Progress in Brain Research*.

[4] Allen M. T., Levy L. S. (2013). Parkinsons disease and pesticide exposure—a new assessment. *Critical Reviews in Toxicology*.

[5] Moretto A., Colosio C. (2013). The role of pesticide exposure in the genesis of Parkinson's disease: epidemiological studies and experimental data. *Toxicology*.

[6] Ono K., Yamada M. (2007). Vitamin A potently destabilizes preformed alpha-synuclein fibrils in vitro: Implications for Lewy body diseases. *Neurobiology of Disease*.

[7] Logroscino G., Marder K., Cote L., Tang M.-X., Shea S., Mayeux R. (1996). Dietary lipids and antioxidants in Parkinson's disease: a population-based, case-control study. *Annals of Neurology*.

[8] Zhang S. M., Hernán M. A., Chen H., Spiegelman D., Willett W. C., Ascherio A. (2002). Intakes of vitamins E and C, carotenoids, vitamin supplements, and PD risk. *Neurology*.

[9] Abbott R. D., Ross G. W., White L. R. (2003). Environmental, life-style, and physical precursors of clinical Parkinson's disease: recent findings from the Honolulu-Asia aging study. *Journal of Neurology*.

[10] Etminan M., Gill S. S., Samii A. (2005). Intake of vitamin E, vitamin C, and carotenoids and the risk of Parkinson's disease: a meta-analysis. *The Lancet Neurology*.

[11] Miyake Y., Fukushima W., Tanaka K. (2011). Dietary intake of antioxidant vitamins and risk of Parkinson's disease: a case-control study in Japan. *European Journal of Neurology*.

[12] Koushik A., Hunter D. J., Spiegelman D. (2006). Intake of the major carotenoids and the risk of epithelial ovarian cancer in a pooled analysis of 10 cohort studies. *International Journal of Cancer*.

[13] Finkelstein J. D. (1990). Methionine metabolism in mammals. *Journal of Nutritional Biochemistry*.

[14] Scott J. M., Weir D. G. (1998). Folic acid, homocysteine and one-carbon metabolism: a review of the essential biochemistry. *Journal of Cardiovascular Risk*.

[15] Duan W., Ladenheim B., Cutler R. G., Kruman I. I., Cadet J. L., Mattson M. P. (2002). Dietary folate deficiency and elevated homocysteine levels endanger dopaminergic neurons in models of Parkinson's disease. *Journal of Neurochemistry*.

[16] Kruman I. I., Culmsee C., Chan S. L. (2000). Homocysteine elicits a DNA damage response in neurons that promotes apoptosis and hypersensitivity to excitotoxicity. *The Journal of Neuroscience*.

[17] Kruman I. I., Kumaravel T. S., Lohani A. (2002). Folic acid deficiency and homocysteine impair DNA repair in hippocampal neurons and sensitize them to amyloid toxicity in experimental models of Alzheimer's disease. *Journal of Neuroscience*.

[18] Adelekan D. A., Thurnham D. I. (1986). Effects of combined riboflavin and iron deficiency on the hematological status and tissue iron concentrations of the rat. *Journal of Nutrition*.

[19] Powers H. J., Weaver L. T., Austin S., Wright A. J. A., Fairweather-Tait S. J. (1991). Riboflavin deficiency in the rat: effects on iron utilization and loss. *British Journal of Nutrition*.

[20] Hellenbrand W., Boeing H., Robra B.-P. (1996). Diet and Parkinson's disease II: a possible role for the past intake of specific nutrients. Results from a self-administered food-frequency questionnaire in a case-control study. *Neurology*.

[21] De Lau L. M. L., Bornebroek M., Witteman J. C. M., Hofman A., Koudstaal P. J., Breteler M. M. B. (2005). Dietary fatty acids and the risk of Parkinson disease: the rotterdam study. *Neurology*.

[22] Murakami K., Miyake Y., Sasaki S. (2010). Dietary intake of folate, vitamin B6, vitamin B12 and riboflavin and risk of Parkinson's disease: a case-control study in Japan. *British Journal of Nutrition*.

[23] Coimbra C. G., Junqueira V. B. C. (2003). High doses of riboflavin and the elimination of dietary red meat promote the recovery of some motor functions in Parkinson's disease patients. *Brazilian Journal of Medical and Biological Research*.

[24] Chen H., Zhang S. M., Schwarzschild M. A. (2004). Folate intake and risk of Parkinson's disease. *The American Journal of Epidemiology*.

[25] Johnson C. C., Gorell J. M., Rybicki B. A., Sanders K., Peterson E. L. (1999). Adult nutrient intake as a risk factor for Parkinson's disease. *International Journal of Epidemiology*.

[26] Seitz G., Gebhardt S., Beck J. F. (1998). Ascorbic acid stimulates DOPA synthesis and tyrosine hydroxylase gene expression in the human neuroblastoma cell line SK-N-SH. *Neuroscience Letters*.

[27] Rice M. E. (2000). Ascorbate regulation and its neuroprotective role in the brain. *Trends in Neurosciences*.

[28] Ghani H., Stevens D., Weiss J., Rosenbaum R. (2002). Vitamins and the risk for Parkinson's disease. *Neurology*.

[29] Eyles D. W., Smith S., Kinobe R., Hewison M., McGrath J. J. (2005). Distribution of the Vitamin D receptor and 1*α*-hydroxylase in human brain. *Journal of Chemical Neuroanatomy*.

[30] Chabas J.-F., Stephan D., Marqueste T. (2013). Cholecalciferol (Vitamin D_3_) improves myelination and recovery after nerve injury. *PLoS ONE*.

[31] Shinpo K., Kikuchi S., Sasaki H., Moriwaka F., Tashiro K. (2000). Effect of 1,25-dihydroxyvitamin D(3) on cultured mesencephalic dopaminergic neurons to the combined toxicity caused by L-buthionine sulfoximine and 1-methyl-4-phenylpyridine. *Journal of Neuroscience Research*.

[32] Wang J.-Y., Wu J.-N., Cherng T.-L. (2001). Vitamin D_3_ attenuates 6-hydroxydopamine-induced neurotoxicity in rats. *Brain Research*.

[33] Sato Y., Kikuyama M., Oizumi K. (1997). High prevalence of vitamin D deficiency and reduced bone mass in Parkinson's disease. *Neurology*.

[34] Sato Y., Kaji M., Tsuru T., Oizumi K. (2001). Risk factors for hip fracture among elderly patients with Parkinson's disease. *Journal of the Neurological Sciences*.

[35] Derex L., Trouillas P. (1997). Reversible Parkinsonism, hypophosphoremia, and hypocalcemia under vitamin D therapy. *Movement Disorders*.

[36] Lv Z., Qi H., Wang L. (2014). Vitamin D status and Parkinson's disease: a systematic review and meta-analysis. *Neurological Sciences*.

[37] Newmark H. L., Newmark J. (2007). Vitamin D and Parkinson's disease: a hypothesis. *Movement Disorders*.

[38] Peterson A. L. (2014). A review of vitamin D and Parkinson's disease. *Maturitas*.

[39] Butler M. W., Burt A., Edwards T. L. (2011). Vitamin D receptor gene as a candidate gene for Parkinson disease. *Annals of Human Genetics*.

[40] Lv Z., Tang B., Sun Q., Yan X., Guo J. (2013). Association study between vitamin D receptor gene polymorphisms and patients with Parkinson disease in Chinese Han population. *International Journal of Neuroscience*.

[41] Lin C.-H., Chen K.-H., Chen M.-L., Lin H.-I., Wu R.-M. (2014). Vitamin D receptor genetic variants and Parkinson's disease in a Taiwanese population. *Neurobiology of Aging*.

[42] Akaneya Y., Takahashi M., Hatanaka H. (1995). Involvement of free radicals in MPP^+^ neurotoxicity against rat dopaminergic neurons in culture. *Neuroscience Letters*.

[43] Dexter D. T., Brooks D. J., Harding A. E. (1994). Nigrostriatal function in vitamin E deficiency: clinical, experimental, and positron emission tomographic studies. *Annals of Neurology*.

[44] Odunze I. N., Klaidman L. K., Adams J. D. (1990). MPTP toxicity in the mouse brain and vitamin E. *Neuroscience Letters*.

[45] Adams J. D., Odunze I. N., Sevanian A. (1990). Induction by 1-methyl-4-phenyl-1,2,3,6-tetrahydropyridine of lipid peroxidation in vivo in vitamin E deficient mice. *Biochemical Pharmacology*.

[46] Abbott R. A., Cox M., Markus H., Tomkins A. (1992). Diet, body size and micronutrient status in Parkinson's disease. *European Journal of Clinical Nutrition*.

[47] Golbe L. I., Farrell T. M., Davis P. H. (1990). Follow-up study of early-life protective and risk factors in Parkinson's disease. *Movement Disorders*.

[48] Morens D. M., Grandinetti A., Waslien C. I., Park C. B., Ross G. W., White L. R. (1996). Case-control study of idiopathic Parkinson's disease and dietary vitamin E intake. *Neurology*.

[49] Shoulson I., Fahn S., Oakes D. (1993). Effects of tocopherol and deprenyl on the progression of disability in early Parkinson's disease. *The New England Journal of Medicine*.

[50] (2000). *Dietary Reference Intakes for Vitamin C, Vitamin E, Selenium, and Carotenoids*.

[51] Olanow C. W. (2003). Dietary vitamin E and Parkinson's disease: something to chew on. *The Lancet Neurology*.

[52] Spencer J. P. E. (2008). Flavonoids: modulators of brain function?. *British Journal of Nutrition*.

[53] Manach C., Scalbert A., Morand C., Remesy C., Jimenez L. (2004). Polyphenols: food sources and bioavailability. *The American Journal of Clinical Nutrition*.

[54] Waterhouse A. L., Shirley J. R., Donovan J. L. (1996). Antioxidants in chocolate. *The Lancet*.

[55] Ramassamy C. (2006). Emerging role of polyphenolic compounds in the treatment of neurodegenerative diseases: a review of their intracellular targets. *European Journal of Pharmacology*.

[56] Yabuki Y., Ohizumi Y., Yokosuka A., Mimaki Y., Fukunaga K. (2014). Nobiletin treatment improves motor and cognitive deficits seen in MPTP-induced parkinson model mice. *Neuroscience*.

[57] Strathearn K. E., Yousef G. G., Grace M. H. (2014). Neuroprotective effects of anthocyanin- and proanthocyanidin-rich extracts in cellular models of Parkinsons disease. *Brain Research*.

[58] Gao X., Cassidy A., Schwarzschild M. A., Rimm E. B., Ascherio A. (2012). Habitual intake of dietary flavonoids and risk of Parkinson disease. *Neurology*.

[59] Sääksjärvi K., Knekt P., Lundqvist A. (2013). A cohort study on diet and the risk of Parkinson's disease: the role of food groups and diet quality. *British Journal of Nutrition*.

[60] Youdim K. A., Qaiser M. Z., Begley D. J., Rice-Evans C. A., Abbott N. J. (2004). Flavonoid permeability across an in situ model of the blood-brain barrier. *Free Radical Biology and Medicine*.

[61] Mattison J. A., Lane M. A., Roth G. S., Ingram D. K. (2003). Calorie restriction in rhesus monkeys. *Experimental Gerontology*.

[62] Weindruch R., Sohal R. S. (1997). Caloric intake and aging. *The New England Journal of Medicine*.

[63] Wanagat J., Allison D. B., Weindruch R. (1999). Caloric intake and aging: mechanisms in rodents and a study in nonhuman primates. *Toxicological Sciences*.

[64] Roth G. S., Joseph J. A. (1994). Cellular and molecular mechanisms of impaired dopaminergic function during aging. *Annals of the New York Academy of Sciences*.

[65] Bruce-Keller A. J., Umberger G., McFall R., Mattson M. P. (1999). Food restriction reduces brain damage and improves behavioral outcome following excitotoxic and metabolic insults. *Annals of Neurology*.

[66] Lee J., Seroogy K. B., Mattson M. P. (2002). Dietary restriction enhances neurotrophin expression and neurogenesis in the hippocampus of adult mice. *Journal of Neurochemistry*.

[67] Duan W., Guo Z., Mattson M. P. (2001). Brain-derived neurotrophic factor mediates an excitoprotective effect of dietary restriction in mice. *Journal of Neurochemistry*.

[68] Mattson M. P. (2003). Excitotoxic and excitoprotective mechanisms: abundant targets for the prevention and treatment of neurodegenerative disorders. *NeuroMolecular Medicine*.

[69] Duan W., Mattson M. P. (1999). Dietary restriction and 2-deoxyglucose administration improve behavioral outcome and reduce degeneration of dopaminergic neurons in models of Parkinson's disease. *Journal of Neuroscience Research*.

[70] Yu Z. F., Mattson M. P. (1999). Dietary restriction and 2-deoxyglucose administration reduce focal ischemic brain damage and improve behavioral outcome: evidence for a preconditioning mechanism. *Journal of Neuroscience Research*.

[71] Jadiya P., Chatterjee M., Sammi S. R., Kaur S., Palit G., Nazir A. (2011). Sir-2.1 modulates “calorie-restriction-mediated” prevention of neurodegeneration in *Caenorhabditis elegans*: implications for Parkinson's disease. *Biochemical and Biophysical Research Communications*.

[72] Armentero M. T., Levandis G., Bramanti P., Nappi G., Blandini F. (2008). Dietary restriction does not prevent nigrostriatal degeneration in the 6-hydroxydopamine model of Parkinson's disease. *Experimental Neurology*.

[73] Maswood N., Young J., Tilmont E. (2004). Caloric restriction increases neurotrophic factor levels and attenuates neurochemical and behavioral deficits in a primate model of Parkinson's disease. *Proceedings of the National Academy of Sciences of the United States of America*.

[74] Mattson M. P. (2003). Will caloric restriction and folate protect against AD and PD?. *Neurology*.

[75] Hernán M. A., Takkouche B., Caamaño-Isorna F., Gestal-Otero J. J. (2002). A meta-analysis of coffee drinking, cigarette smoking, and the risk of Parkinson's disease. *Annals of Neurology*.

[76] Ross G. W., Abbott R. D., Petrovitch H., White L. R., Tanner C. M. (2000). Relationship between caffeine intake and Parkinson disease. *Journal of American Medical Association*.

[77] Kyrozis A., Ghika A., Stathopoulos P., Vassilopoulos D., Trichopoulos D., Trichopoulou A. (2013). Dietary and lifestyle variables in relation to incidence of Parkinson's disease in Greece. *European Journal of Epidemiology*.

[78] Popoli P., Caporali M. G., de Carolis A. S. (1991). Akinesia due to catecholamine depletion in mice is prevented by caffeine. Further evidence for an involvement of adenosinergic system in the control of motility. *Journal of Pharmacy and Pharmacology*.

[79] Kanda T., Tashiro T., Kuwana Y., Jenner P. (1998). Adenosine A2A receptors modify motor function in MPTP-treated common marmosets. *NeuroReport*.

[80] Richardson P. J., Kase H., Jenner P. G. (1997). Adenosine A_2A_ receptor antagonists as new agents for the treatment of Parkinson's disease. *Trends in Pharmacological Sciences*.

[81] Chen J. F., Xu K., Petzer J. P. (2001). Neuroprotection by caffeine and A_2A_ adenosine receptor inactivation in a model of Parkinson's disease. *The Journal of Neuroscience*.

[82] Nehlig A., Daval J.-L., Debry G. (1992). Caffeine and the central nervous system: mechanisms of action, biochemical, metabolic and psychostimulant effects. *Brain Research Reviews*.

[83] Ascherio A., Zhang S. M., Hernán M. A. (2001). Prospective study of caffeine consumption and risk of Parkinson's disease in men and women. *Annals of Neurology*.

[84] Ascherio A., Weisskopf M. G., O'Reilly E. J. (2004). Coffee consumption, gender, and Parkinson's disease mortality in the cancer prevention study II cohort: the modifying effects of estrogen. *American Journal of Epidemiology*.

[85] Grandinetti A., Morens D. M., Reed D., MacEachern D. (1994). Prospective study of cigarette smoking and the risk of developing idiopathic Parkinson's disease. *American Journal of Epidemiology*.

[86] Ross G. W., Abbott R. D., Petrovitch H. (2000). Association of coffee and caffeine intake with the risk of Parkinson disease. *The Journal of the American Medical Association*.

[87] Liu R., Guo X., Park Y. (2012). Caffeine intake, smoking, and risk of parkinson disease in men and women. *American Journal of Epidemiology*.

[88] Tan L. C., Koh W.-P., Yuan J.-M. (2008). Differential effects of black versus green tea on risk of Parkinson's disease in the Singapore Chinese health study. *American Journal of Epidemiology*.

[89] Chan D. K. Y., Woo J., Ho S. C. (1998). Genetic and environmental risk factors for Parkinson's disease in a Chinese population. *Journal of Neurology Neurosurgery and Psychiatry*.

[90] Checkoway H., Powers K., Smith-Weller T., Franklin G. M., Longstreth W. T., Swanson P. D. (2002). Parkinson's disease risks associated with cigarette smoking, alcohol consumption, and caffeine intake. *American Journal of Epidemiology*.

[91] Tan E.-K., Tan C., Fook-Chong S. M. C. (2003). Dose-dependent protective effect of coffee, tea, and smoking in Parkinson's disease: a study in ethnic Chinese. *Journal of the Neurological Sciences*.

[92] Powers K. M., Kay D. M., Factor S. A. (2008). Combined effects of smoking, coffee, and NSAIDs on Parkinson's disease risk. *Movement Disorders*.

[93] Rugbjerg K., Christensen J., Tjønneland A., Olsen J. H. (2013). Exposure to estrogen and women's risk for Parkinson's disease: a prospective cohort study in Denmark. *Parkinsonism & Related Disorders*.

[94] Hu G., Bidel S., Jousilahti P., Antikainen R., Tuomilehto J. (2007). Coffee and tea consumption and the risk of Parkinson's disease. *Movement Disorders*.

[95] Ascherio A., Chen H., Schwarzschild M. A., Zhang S. M., Colditz G. A., Speizer F. E. (2003). Caffeine, postmenopausal estrogen, and risk of Parkinson's disease. *Neurology*.

[96] Taylor H. S., Manson J. E. (2011). Update in hormone therapy use in menopause. *Journal of Clinical Endocrinology and Metabolism*.

[97] Bowen R. A. R., Clandinin M. T. (2002). Dietary low linolenic acid compared with docosahexaenoic acid alter synaptic plasma membrane phospholipid fatty acid composition and sodium-potassium ATPase kinetics in developing rats. *Journal of Neurochemistry*.

[98] Ikemoto A., Ohishi M., Sato Y. (2001). Reversibility of n-3 fatty acid deficiency-induced alterations of learning behavior in the rat: level of n-6 fatty acids as another critical factor. *Journal of Lipid Research*.

[99] Levant B., Ozias M. K., Carlson S. E. (2007). Specific brain regions of female rats are differentially depleted of docosahexaenoic acid by reproductive activity and an (n-3) fatty acid-deficient diet. *Journal of Nutrition*.

[100] Logan A. C. (2004). Omega-3 fatty acids and major depression: a primer for the mental health professional. *Lipids in Health and Disease*.

[101] Moriguchi T., Greiner R. S., Salem N. (2000). Behavioral deficits associated with dietary induction of decreased brain docosahexaenoic acid concentration. *Journal of Neurochemistry*.

[102] Farooqui A. A., Horrocks L. A., Farooqui T. (2000). Glycerophospholipids in brain: their metabolism, incorporation into membranes, functions, and involvement in neurological disorders. *Chemistry and Physics of Lipids*.

[103] Youdim K. A., Martin A., Joseph J. A. (2000). Essential fatty acids and the brain: possible health implications. *International Journal of Developmental Neuroscience*.

[104] Wu A., Molteni R., Ying Z., Gomez-Pinilla F. (2003). A saturated-fat diet aggravates the outcome of traumatic brain injury on hippocampal plasticity and cognitive function by reducing brain-derived neurotrophic factor. *Neuroscience*.

[105] Calon F., Lim G. P., Yang F. (2004). Docosahexaenoic acid protects from dendritic pathology in an Alzheimer's disease mouse model. *Neuron*.

[106] de Smedt-Peyrusse V., Sargueil F., Moranis A., Harizi H., Mongrand S., Layé S. (2008). Docosahexaenoic acid prevents lipopolysaccharide-induced cytokine production in microglial cells by inhibiting lipopolysaccharide receptor presentation but not its membrane subdomain localization. *Journal of Neurochemistry*.

[107] Hashimoto M., Hossain S., Shimada T. (2002). Docosahexaenoic acid provides protection from impairment of learning ability in Alzheimer's disease model rats. *Journal of Neurochemistry*.

[108] Hashimoto M., Tanabe Y., Fujii Y., Kikuta T., Shibata H., Shido O. (2005). Chronic administration of docosahexaenoic acid ameliorates the impairment of spatial cognition learning ability in amyloid *β*-infused rats. *Journal of Nutrition*.

[109] Schapira A. H. V. (2007). Mitochondrial dysfunction in Parkinson's disease. *Cell Death and Differentiation*.

[110] Wu A., Ying Z., Gomez-Pinilla F. (2004). Dietary omega-3 fatty acids normalize BDNF levels, reduce oxidative damage, and counteract learning disability after traumatic brain injury in rats. *Journal of Neurotrauma*.

[111] Fernández-Ruiz J., Lastres-Becker I., Cabranes A., González S., Ramos J. A. (2002). Endocannabinoids and basal ganglia functionality. *Prostaglandins Leukotrienes and Essential Fatty Acids*.

[112] Giuffrida A., Piomelli D. (2000). The endocannabinoid system: a physiological perspective on its role in psychomotor control. *Chemistry and Physics of Lipids*.

[113] Brodoehl S., Klingner C., Volk G. F., Bitter T., Witte O. W., Redecker C. (2012). Decreased olfactory bulb volume in idiopathic Parkinson's disease detected by 3.0-Tesla magnetic resonance imaging. *Movement Disorders*.

[114] Liberini P., Parola S., Spano P. F., Antonini L. (2000). Olfaction in Parkinson's disease: methods of assessment and clinical relevance. *Journal of Neurology*.

[115] Zimmer L., Vancassel S., Cantagrel S. (2002). The dopamine mesocorticolimbic pathway is affected by deficiency in n-3 polyunsaturated fatty acids. *American Journal of Clinical Nutrition*.

[116] Samadi P., Grégoire L., Rouillard C., Bédard P. J., Di Paolo T., Lévesque D. (2006). Docosahexaenoic acid reduces levodopa-induced dyskinesias in 1-methyl-4-phenyl-1,2,3,6-tetrahydropyridine monkeys. *Annals of Neurology*.

[117] Delattre A. M., Kiss Á., Szawka R. E. (2010). Evaluation of chronic omega-3 fatty acids supplementation on behavioral and neurochemical alterations in 6-hydroxydopamine-lesion model of Parkinson's disease. *Neuroscience Research*.

[118] Bousquet M., Saint-Pierre M., Julien C., Salem N., Cicchetti F., Calon F. (2008). Beneficial effects of dietary omega-3 polyunsaturated fatty acid on toxin-induced neuronal degeneration in an animal model of Parkinson's disease. *The FASEB Journal*.

[119] Assayag K., Yakunin E., Loeb V., Selkoe D. J., Sharon R. (2007). Polyunsaturated fatty acids induce *α*-synuclein-related pathogenic changes in neuronal cells. *The American Journal of Pathology*.

[120] Broersen K., van den Brink D., Fraser G., Goedert M., Davletov B. (2006). *α*-synuclein adopts an *α*-helical conformation in the presence of polyunsaturated fatty acids to hinder micelle formation. *Biochemistry*.

[121] Sharon R., Bar-Joseph I., Frosch M. P., Walsh D. M., Hamilton J. A., Selkoe D. J. (2003). The formation of highly soluble oligomers of *α*-synuclein is regulated by fatty acids and enhanced in Parkinson's disease. *Neuron*.

[122] Barceló-Coblijn G., Kitajka K., Puskás L. G. (2003). Gene expression and molecular composition of phospholipids in rat brain in relation to dietary n-6 to n-3 fatty acid ratio. *Biochimica et Biophysica Acta: Molecular and Cell Biology of Lipids*.

[123] Kitajka K., Sinclair A. J., Weisinger R. S. (2004). Effects of dietary omega-3 polyunsaturated fatty acids on brain gene expression. *Proceedings of the National Academy of Sciences of the United States of America*.

[124] Yakunin E., Loeb V., Kisos H. (2012). *α*-Synuclein neuropathology is controlled by nuclear hormone receptors and enhanced by docosahexaenoic acid in a mouse model for Parkinson's disease. *Brain Pathology*.

[125] Bruce-Keller A. J., Keller J. N., Morrison C. D. (2009). Obesity and vulnerability of the CNS. *Biochimica et Biophysica Acta*.

[126] Morris J. K., Bomhoff G. L., Stanford J. A., Geiger P. C. (2010). Neurodegeneration in an animal model of Parkinson's disease is exacerbated by a high-fat diet. *The American Journal of Physiology: Regulatory Integrative and Comparative Physiology*.

[127] Morris J. K., Bomhoff G. L., Gorres B. K. (2011). Insulin resistance impairs nigrostriatal dopamine function. *Experimental Neurology*.

[128] Chen H., Zhang S. M., Hernán M. A., Willett W. C., Ascherio A. (2003). Dietary intakes of fat and risk of Parkinson's disease. *American Journal of Epidemiology*.

[129] Hellenbrand W., Seidler A., Boeing H. (1996). Diet and Parkinson's disease I: a possible role for the past intake of specific foods and food groups. Results from a self-administered food- frequency questionnaire in a case-control study. *Neurology*.

[130] Powers K. M., Smith-Weller T., Franklin G. M., Longstreth W. T., Swanson P. D., Checkoway H. (2003). Parkinson's disease risks associated with dietary iron, manganese, and other nutrient intakes. *Neurology*.

[131] da Silva T. M., Munhoz R. P., Alvarez C. (2008). Depression in Parkinson's disease: a double-blind, randomized, placebo-controlled pilot study of omega-3 fatty-acid supplementation. *Journal of Affective Disorders*.

[132] Kamel F., Goldman S. M., Umbach D. M. (2014). Dietary fat intake, pesticide use, and Parkinson's disease. *Parkinsonism & Related Disorders*.

[133] Anderson C., Checkoway H., Franklin G. M., Beresford S., Smith-Weller T., Swanson P. D. (1999). Dietary factors in Parkinson's disease: the role of food groups and specific foods. *Movement Disorders*.

[134] Dong J., Beard J. D., Umbach D. M. (2014). Dietary fat intake and risk for Parkinson's disease. *Movement Disorders*.

[135] Oliveira A., Cacodcar J., Motghare D. D. (2014). Morbidity among iron ore mine workers in Goa. *Indian Journal of Public Health*.

[136] Mortimer J. A., Borenstein A. R., Nelson L. M. (2012). Associations of welding and manganese exposure with Parkinson disease: review and meta-analysis. *Neurology*.

[137] Gerlach M., Ben-Shachar D., Riederer P., Youdim M. B. H. (1994). Altered brain metabolism of iron as a cause of neurodegenerative diseases?. *Journal of Neurochemistry*.

[138] Egaña J. T., Zambrano C., Nuñez M. T., Gonzalez-Billault C., Maccioni R. B. (2003). Iron-induced oxidative stress modify tau phosphorylation patterns in hippocampal cell cultures. *BioMetals*.

[139] Ke Y., Ming Qian Z. (2003). Iron misregulation in the brain: a primary cause of neurodegenerative disorders. *The Lancet Neurology*.

[140] Zecca L., Youdim M. B. H., Riederer P., Connor J. R., Crichton R. R. (2004). Iron, brain ageing and neurodegenerative disorders. *Nature Reviews Neuroscience*.

[141] Castellani R. J., Siedlak S. L., Perry G., Smith M. A. (2000). Sequestration of iron by Lewy bodies in Parkinson's disease. *Acta Neuropathologica*.

[142] Golts N., Snyder H., Frasier M., Theisler C., Choi P., Wolozin B. (2002). Magnesium inhibits spontaneous and iron-induced aggregation of *α*-synuclein. *The Journal of Biological Chemistry*.

[143] Hirsch E. C., Breidert T., Rousselet E., Hunot S., Hartmann A., Michel P. P. (2003). The role of glial reaction and inflammation in Parkinson's disease. *Annals of the New York Academy of Sciences*.

[144] Zhang X., Surguladze N., Slagle-Webb B., Cozzi A., Connor J. R. (2006). Cellular iron status influences the functional relationship between microglia and oligodendrocytes. *GLIA*.

[145] Lan J., Jiang D. H. (1997). Excessive iron accumulation in the brain: a possible potential risk of neurodegeneration in Parkinson's disease. *Journal of Neural Transmission*.

[146] Kaur D., Yantiri F., Rajagopalan S. (2003). Genetic or pharmacological iron chelation prevents MPTP-induced neurotoxicity in vivo: a novel therapy for Parkinson's disease. *Neuron*.

[147] Ostrerova-Golts N., Petrucelli L., Hardy J., Lee J. M., Farer M., Wolozin B. (2000). The A53T *α*-synuclein mutation increases iron-dependent aggregation and toxicity. *The Journal of Neuroscience*.

[148] Erikson K. M., Jones B. C., Beard J. L. (2000). Iron deficiency alters dopamine transporter functioning in rat striatum. *Journal of Nutrition*.

[149] Beard J., Erikson K. M., Jones B. C. (2003). Neonatal iron deficiency results in irreversible changes in dopamine function in rats. *Journal of Nutrition*.

[150] Beard J. L., Felt B., Schallert T., Burhans M., Connor J. R., Georgieff M. K. (2006). Moderate iron deficiency in infancy: biology and behavior in young rats. *Behavioural Brain Research*.

[151] Burhans M. S., Dailey C., Wiesinger J., Murray-Kolb L. E., Jones B. C., Beard J. L. (2006). Iron deficiency affects acoustic startle response and latency, but not prepulse inhibition in young adult rats. *Physiology & Behavior*.

[152] Levenson C. W., Cutler R. G., Ladenheim B., Cadet J. L., Hare J., Mattson M. P. (2004). Role of dietary iron restriction in a mouse model of Parkinson's disease. *Experimental Neurology*.

[153] Jiang H., Luan Z., Wang J., Xie J. (2006). Neuroprotective effects of iron chelator Desferal on dopaminergic neurons in the substantia nigra of rats with iron-overload. *Neurochemistry International*.

[154] Logroscino G., Marder K., Graziano J. (1998). Dietary iron, animal fats, and risk of Parkinson's disease. *Movement Disorders*.

[155] Checkoway H., Nelson L. M. (1999). Epidemiologic approaches to the study of Parkinson's disease etiology. *Epidemiology*.

[156] Logroscino G., Gao X., Chen H., Wing A., Ascherio A. (2008). Dietary iron intake and risk of Parkinson's disease. *The American Journal of Epidemiology*.

[157] Fukushima T., Tan X., Luo Y., Kanda H. (2010). Relationship between blood levels of heavy metals and Parkinson's disease in China. *Neuroepidemiology*.

[158] Borie C., Gasparini F., Verpillat P. (2002). Association study between iron-related genes polymorphisms and Parkinson's disease. *Journal of Neurology*.

[159] Dekker M. C. J., Giesbergen P. C., Njajou O. T. (2003). Mutations in the hemochromatosis gene (HFE), Parkinson's disease and parkinsonism. *Neuroscience Letters*.

[160] Mastroberardino P. G., Hoffman E. K., Horowitz M. P. (2009). A novel transferrin/TfR2-mediated mitochondrial iron transport system is disrupted in Parkinson's disease. *Neurobiology of Disease*.

[161] Takeda A. (2003). Manganese action in brain function. *Brain Research Reviews*.

[162] Gorell J. M., Johnson C. C., Rybicki B. A. (1997). Occupational exposures to metals as risk factors for Parkinson's disease. *Neurology*.

[163] Williams M., Todd G. D., Roney N. (2012). *Toxicological Profile for Manganese*.

[164] Chua A. C. G., Morgan E. H. (1996). Effects of iron deficiency and iron overload on manganese uptake and deposition in the brain and other organs of the rat. *Biological Trace Element Research*.

[165] Saris N.-E. L., Mervaala E., Karppanen H., Khawaja J. A., Lewenstam A. (2000). Magnesium: an update on physiological, clinical and analytical aspects. *Clinica Chimica Acta*.

[166] Hashimoto T., Nishi K., Nagasao J., Tsuji S., Oyanagi K. (2008). Magnesium exerts both preventive and ameliorating effects in an in vitro rat Parkinson disease model involving 1-methyl-4-phenylpyridinium (MPP+) toxicity in dopaminergic neurons. *Brain Research*.

[167] Nakamura K., Bindokas V. P., Marks J. D. (2000). The selective toxicity of 1-methyl-4-phenylpyridinium to dopaminergic neurons: the role of mitochondrial complex I and reactive oxygen species revisited. *Molecular Pharmacology*.

[168] Oyanagi K., Kawakami E., Kikuchi-Horie K. (2006). Magnesium deficiency over generations in rats with special references to the pathogenesis of the parkinsonism-dementia complex and amyotrophic lateral sclerosis of Guam. *Neuropathology*.

[169] Taniguchi R., Nakagawasai O., Tan-No K. (2013). Combined low calcium and lack magnesium is a risk factor for motor deficit in mice. *Bioscience, Biotechnology and Biochemistry*.

[170] Mayer M. L., Westbrook G. L., Buthrie P. B. (1984). Voltage-dependent block by Mg^2+^ of NMDA responses in spinal cord neurones. *Nature*.

[171] Aden E., Carlsson M., Poortvliet E. (2011). Dietary intake and olfactory function in patients with newly diagnosed parkinson's disease: a case-control study. *Nutritional Neuroscience*.

[172] Bharucha N. E., Stokes L., Schoenberg B. S. (1986). A case-control study of twin pairs discordant for Parkinson's disease: a search for environmental risk factors. *Neurology*.

[173] Jimenez-Jimenez F. J., Mateo D., Gimenez-Roldan S. (1992). Premorbid smoking, alcohol consumption, and coffee drinking habits in Parkinson's disease: a case-control study. *Movement Disorders*.

[174] Fall P. A., Fredrikson M., Axelson O., Granerus A. K. (1999). Nutritional and occupational factors influencing the risk of Parkinson's disease: a case-control study in southeastern Sweden. *Movement Disorders*.

[175] Paganini-Hill A. (2001). Risk factors for Parkinson's disease: the Leisure World cohort study. *Neuroepidemiology*.

[176] Ragonese P., Salemi G., Morgante L. (2003). A case-control study on cigarette, alcohol, and coffee consumption preceding Parkinson's disease. *Neuroepidemiology*.

[177] Eriksson A.-K., Löfving S., Callaghan R. C., Allebeck P. (2013). Alcohol use disorders and risk of Parkinson's disease: findings from a Swedish national cohort study 1972–2008. *BMC Neurology*.

[178] Zhang D., Jiang H., Xie J. (2014). Alcohol intake and risk of Parkinson's disease: a meta-analysis of observational studies. *Movement Disorders*.

[179] Felton J. S., Knize M. G., Wood C. (1984). Isolation and characterization of new mutagens from fried ground beef. *Carcinogenesis*.

[180] Turesky R. J. (2007). Formation and biochemistry of carcinogenic heterocyclic aromatic amines in cooked meats. *Toxicology Letters*.

[181] Shioya M., Wakabayashi K., Sato S., Nagao M., Sugimura T. (1987). Formation of a mutagen, 2-amino-1-methyl-6-phenylimidazo[4,5-b]-pyridine (PhIP) in cooked beef, by heating a mixture containing creatinine, phenylalanine and glucose. *Mutation Research Letters*.

[182] Snyderwine E. G., Buonarati M. H., Felton J. S., Turteltaub K. W. (1993). Metabolism of the food-derived mutagen/carcinogen 2-amino-1-methyl-6-phenylimidazo[4,5-b]pyridine (PhIP) in nonhuman primates. *Carcinogenesis*.

[183] Felton J. S., Knize M. G. (1991). Occurrence, identification, and bacterial mutagenicity of heterocyclic amines in cooked food. *Mutation Research*.

[184] Felton J. S., Knize M. G., Wu R. W., Colvin M. E., Hatch F. T., Malfatti M. A. (2007). Mutagenic potency of food-derived heterocyclic amines. *Mutation Research: Fundamental and Molecular Mechanisms of Mutagenesis*.

[185] Hiroshi I., Norio O., Daiichirou N. (1988). Effects of heterocyclic amines in food on dopamine metabolism in nigro-striatal dopaminergic neurons. *Biochemical Pharmacology*.

[186] Kojima T., Naoi M., Wakabayashi K., Sugimura T., Nagatsu T. (1990). 3-amino-1-methyl-5H-pyrido[4,3-b]indole (Trp-P-2) and other heterocyclic amines as inhibitors of mitochondrial monoamine oxidases separated from human brain synaptosomes. *Neurochemistry International*.

[187] Maruyama W., Ota A., Takahashi A., Nagatsu T., Naoi M. (1994). Food-derived heterocyclic amines, 3-amino-1,4-dimethyl-5H-pyrido[4,3-b]indole and related amines, as inhibitors of monoamine metabolism. *Journal of Neural Transmission*.

[188] Ota M., Naoi M., Hamanaka T., Nagatsu T. (1990). Inhibition of human brain aromatic l-amino acid decarboxylase by cooked food-derived 3-amino-1-methyl-5H-pyrido[4,3-b]indole (Trp-P-2) and other heterocyclic amines. *Neuroscience Letters*.

[189] Felton J. S., Knize M. G., Shen N. H., Andresen B. D., Bjeldanes L. F., Hatch F. T. (1986). Identification of the mutagens in cooked beef. *Environmental Health Perspectives*.

[190] Felton J. S., Knize M. G., Shen N. H. (1986). The isolation and identification of a new mutagen from fried ground beef: 2-amino-1-methyl-6-phenylimidazo[4,5-b]pyridine (PhIP). *Carcinogenesis*.

[191] Teunissen S. F., Vlaming M. L. H., Rosing H., Schellens J. H. M., Schinkel A. H., Beijnen J. H. (2010). Development and validation of a liquid chromatography-tandem mass spectrometry assay for the analysis of 2-amino-1-methyl-6-phenylimidazo[4,5-b]pyridine (PhIP) and its metabolite 2-hydroxyamino-1-methyl-6-phenylimidazo[4,5-b]pyridine (N-OH-PhIP) in plasma, urine, bile, intestinal contents, faeces and eight selected tissues from mice. *Journal of Chromatography B: Analytical Technologies in the Biomedical and Life Sciences*.

[192] Griggs A. M., Agim Z. S., Mishra V. R. (2014). 2-Amino-1-methyl-6-phenylimidazo[4,5-b]pyridine (PhIP) is selectively toxic to primary dopaminergic neurons in vitro. *Toxicological Sciences*.

[193] Pfau W., Skog K. (2004). Exposure to *β*-carbolines norharman and harman. *Journal of Chromatography B: Analytical Technologies in the Biomedical and Life Sciences*.

[194] Benito-Leon J., Bermejo-Pareja F., Morales J. M., Vega S., Molina J.-A. (2003). Prevalence of essential tremor in three elderly populations of central Spain. *Movement Disorders*.

[195] Dogu O., Sevim S., Camdeviren H. (2003). Prevalence of essential tremor: door-to-door neurologic exams in Mersin Province, Turkey. *Neurology*.

[196] Louis E. D., Jiang W., Pellegrino K. M. (2008). Elevated blood harmane (1-methyl-9H-pyrido[3,4-b]indole) concentrations in essential tremor. *NeuroToxicology*.

[197] Louis E. D., Factor-Litvak P., Gerbin M. (2011). Blood harmane, blood lead, and severity of hand tremor: evidence of additive effects. *NeuroToxicology*.

[198] Louis E. D., Michalec M., Jiang W., Factor-Litvak P., Zheng W. (2014). Elevated blood harmane (1-methyl-9H-pyrido[3,4-b]indole) concentrations in Parkinson's disease. *NeuroToxicology*.

[199] Sienkiewicz-Jarosz H., Scinska A., Swiecicki L. (2013). Sweet liking in patients with Parkinson's disease. *Journal of the Neurological Sciences*.

[200] Miwa H., Kondo T. (2008). Alteration of eating behaviors in patients with Parkinson's disease: Possibly overlooked?. *Neurocase*.

[201] Jiang W., Ju C., Jiang H., Zhang D. (2014). Dairy foods intake and risk of Parkinson's disease: a dose-response meta-analysis of prospective cohort studies. *European Journal of Epidemiology*.

[202] Willett W. C., Sacks F., Trichopoulou A. (1995). Mediterranean diet pyramid: a cultural model for healthy eating. *The American Journal of Clinical Nutrition*.

[203] Alcalay R. N., Gu Y., Mejia-Santana H., Cote L., Marder K. S., Scarmeas N. (2012). The association between Mediterranean diet adherence and Parkinson's disease. *Movement Disorders*.

[204] Yamada-Fowler N., Fredrikson M., Soderkvist P. (2014). Caffeine interaction with glutamate receptor gene GRIN2A: Parkinson's disease in Swedish population. *PloS ONE*.

[205] Hamza T. H., Chen H., Hill-Burns E. M. (2011). Genome-wide gene-environment study identifies glutamate receptor gene GRIN2A as a parkinson's disease modifier gene via interaction with coffee. *PLoS Genetics*.

